# Risk factors of immune checkpoint inhibitor-related pneumonitis after neoadjuvant immunochemotherapy for resectable NSCLC

**DOI:** 10.1186/s12890-024-03041-6

**Published:** 2024-05-23

**Authors:** Zhirong Mao, Guanchao Pang, Xiaojie Huang, Xiuxiu Chen, Jiaji Wu, Xia Xu, Zhihua Teng, Yanbin Tan, Pingli Wang

**Affiliations:** 1 Department of Respiratory Medicine, School of Medicine, Second Affiliated Hospital of Zhejiang University, Hangzhou, 310000 Zhejiang China; 2 Department of Respiratory and Critical Care Medicine, The Fourth Affiliated Hospital, International Institutes of Medicine, Zhejiang University School of Medicine, Yiwu, 322000 Zhejiang China; 3 Department of Pathology, School of Medicine, Second Affiliated Hospital of Zhejiang University, Hangzhou, 310000 Zhejiang China; 4 Department of Thoracic Surgery, School of Medicine, Second Affiliated Hospital of Zhejiang University, Hangzhou, 310000 Zhejiang China; 5 Department of Radiology, School of Medicine, Second Affiliated Hospital of Zhejiang University, Hangzhou, 310000 Zhejiang China

**Keywords:** Non-small cell lung cancer, Checkpoint inhibitor-associated pneumonitis, Body mass index, Body mass index

## Abstract

**Background:**

The incidence of checkpoint inhibitor-associated pneumonitis (CIP) in advanced non-small cell lung cancer (NSCLC) has been substantiated through large-scale clinical trials or real-world studies. However, reports on CIP incidence within the context of neoadjuvant immunotherapy for resectable NSCLC remain scarce. This study endeavors to investigate the incidence, risk factors, and outcomes of CIP in patients with resectable NSCLC receiving neoadjuvant immunochemotherapy.

**Methods:**

A retrospective, case-control study was conducted on patients diagnosed with NSCLC stages IIA–IIIB who received neoadjuvant immunochemotherapy between January 2018 and September 2022. Patients were stratified into two groups based on the presence or absence of CIP, facilitating a comparative analysis of clinical characteristics, treatment modalities, physiological indicators, and prognostic outcomes .

**Results:**

The study cohort comprised 245 patients, with 11.4% (28/245) experiencing CIP. The median period of CIP onset was 70 (range, 40–221) days. The incidence of severe CIP (grade 3–4) was 3.7% (9/245). Patients with CIP showed a higher all-cause mortality rate of 21.4% (6/28) compared to that of patients without CIP. Those who developed CIP exhibited elevated body mass index (BMI) values (*p* = 0.028) and increased fibrinogen (FIB) levels (*p* < 0.001), alongside a significant decrease in both diffusing capacity for carbon monoxide (DLCO)% pred (*p* = 0.001) and DLCO/VA% pred (*p* = 0.021) after neoadjuvant therapy compared to pre-indicators. Receiver operating characteristic curve (ROC) analysis showed that the area under the ROC curve of three assessed variables (FIB levels, BMI, DLCO) reached 0.806 in predicting CIP occurrence at an early stage.

**Conclusions:**

This cohort demonstrated that elevated BMI, increased FIB levels, and decreased pulmonary diffusion function after neoadjuvant therapy are risk factors of CIP occurrence. Early assessment and continuous monitoring of these indicators are imperative for the predictive identification of CIP, enhancing patient management and outcomes.

## Introduction

The implementation of immune checkpoint inhibitors (ICIs), specifically programmed cell death protein 1 (PD-1)/ PD receptor ligand-1 (PD-L1) antibodies, has significantly improved the progression-free-survival (PFS) and overall survival (OS) rates of patients with advanced non-small cell lung carcinoma (NSCLC) [1, 2]. In early-stage NSCLC, the application of PD-1/PD-L1 antibodies has similarly demonstrated survival benefits. Evidence from multiple studies indicates that neoadjuvant immunotherapy or immunochemotherapy significantly increased the incidence rate of complete pathological reactions (pCR) and major pathological reactions (MPR), contributing to an increase in the 5-year OS rate from 40 to 85% [3–5]. Neoadjuvant immunochemotherapy has been verified to be the most effective way to increase the pCR and MPR rates and reduce the risk of postoperative recurrence, which has become the preferred strategy for neoadjuvant therapy.

Apart from improving the prognosis of patients with NSCLC, ICIs also cause a variety of immune-related adverse events (irAEs), including but not limited to rash, myocarditis, hepatitis, colitis, hypothyroidism, nephritis, and pneumonitis [6]. Among these, Checkpoint Inhibitor-Associated Pneumonitis (CIP) represents a relatively rare yet potentially lethal irAE. Several large-scale clinical trials have reported CIP incidence rates ranging from 2 to 5% in advanced NSCLC cases [7, 8], with real-world investigations indicating considerably higher prevalence rates [9–11]. However, there are few reports of CIP in neoadjuvant immunotherapy for resectable NSCLC. The stress reaction caused by surgical trauma results in immunological effects that presumably increase the occurrence of CIP.

Understanding the incidence, potential risk factors, and survival outcomes associated with CIP in patients undergoing neoadjuvant immunochemotherapy is crucial to improving patient care quality. This study primarily focused on delineating the frequency and determinants of CIP within a real-world cohort subjected to neoadjuvant immunochemotherapy. Emphasizing the analysis of clinical data collected both before and after neoadjuvant immunotherapy, special attention was devoted to evaluating outcomes from pulmonary function tests. Through these evaluations, the research aimed to stratify the risk of CIP development, thereby enabling the proactive identification of patients at heightened risk for severe manifestations of CIP.

## Methods

### Study population and data source

This study enrolled patients diagnosed with NSCLC stages IIA–IIIB who underwent neoadjuvant immunochemotherapy prior to surgical intervention at the Second Affiliated Hospital of Zhejiang University between January 2018 and September 2022. The demographic, clinical, and laboratory test data were obtained via electronic medical record systems. Follow-up data and survival information were obtained from outpatient reviews or telephone interviews. The institutional review board of the Second Affiliated Hospital of Zhejiang University granted approval for this retrospective study (approval number: 2022 − 0569), and informed consent was obtained in adherence to the Declaration of Helsinki.

### Inclusion criteria and CIP evaluation

All patients who were registered underwent a minimum 1-month postoperative follow-up period and at least three chest computed tomography (CT) examinations. The whole examinations comprised routine follow-up prior to and following neoadjuvant immunochemotherapy, as well as postoperative visits. None of the patients received preoperative radiotherapy or target therapy. Most selected patients received chemotherapy of platinum-based combinations, predominantly paclitaxel or pemetrexed. All patients were treated with ≥ 1 dose of a PD-1/PD-L1 inhibitor (Sintilimab, Tislelizumab, Toripalimab, Pembrolizumab, Camrelizumab, Nivolumab, or Durvalumab). Depending on the outcomes of neoadjuvant immunochemotherapy, surgery is carried out after 1–4 cycles of combination treatment. Prior to neoadjuvant therapy, all enrolled patients underwent medical history assessment and routine blood tests. Additionally, they underwent lung function tests and chest CT scans both before and after the neoadjuvant therapy, and received a minimum of one postoperative lung CT follow-up examination.

In accordance with the expert consensus from China on CIP [12], the diagnostic criteria for this condition involve a documented history of ICIs usage, followed by the emergence of specific clinical symptoms such as cough, chest pain, fever, or hypoxemia. Additionally, it is imperative to demonstrate the emergence of novel pulmonary abnormalities via imaging modalities, whilst meticulously excluding other plausible differential diagnoses such as pulmonary infections, tumor progression, pulmonary edema, and thromboembolic events. Referring to the guidelines established by the American Society of Clinical Oncology (ASCO) in 2018 regarding immune-related adverse events (irAEs), CIP was graded into four categories [13]: G1- Asymptomatic, confined to one lobe of the lung or < 25% of lung parenchyma, clinical or diagnostic observations only; G2- Symptomatic, involving more than one lobe of the lung or 25–50% of lung parenchyma, medical intervention indicated, limiting instrumental activities of daily living (ADLs); G3- Severe symptoms, hospitalization required, involving all lung lobes or 50% of lung parenchyma, limiting self-care ADLs, oxygen indicated; and G4- Life-threatening respiratory compromise, urgent intervention indicated (intubation).

### Data collection

Clinical and biological characteristics of the study population, including demographics, sex, age, comorbidities, tumor histologic type, preoperative tumor node metastasis (TNM) stage, postoperative pathologic response, neoadjuvant therapy strategies, types of ICIs, and outcomes of CIP: pre-treatment pulmonary CT abnormalities (normal, bronchitis/emphysema, proliferative lesions, atelectasis, bronchiectasis, interstitial lung abnormality). Physiological and biochemical characteristics, including body mass index (BMI), C-reactive protein (CRP), fasting blood glucose (FBG), white blood cell (WBC) count, neutrophil-lymphocyte ratio (NLR), fibrinogen (FIB) levels, lactate dehydrogenase (LDH), vitalcapacity (VC), peak expiratory flow (PEF), peak inspiratory flow (PIF), forced expiratory volume in 1 s/ forced vital capacity ratio (FEV1/FVC%), diffusing capacity of the lungs for carbon monoxide/predicted values (DLCO% predicted), and DLCO by alveolar volume/predicted values (DLCO/VA% predicted), were recorded before and after 1–4 cycles of neoadjuvant immunochemotherapy. The spirometry test results were normalized by expressing them as a percentage of the predicted value (% of predicted), which was calculated by dividing the actual pulmonary function test values by the predicted values.

Chest CT images of all patients were reviewed at presentation and at every follow-up examination. To identify patients with CIP, suspicious images were reviewed by a panel consisting of three senior respiratory physicians and one senior radiologist. The panel’s decision regarding CIP diagnosis was based on their review of the suspicious images. The expert panel also made reference to the patient’s subsequent diagnosis, treatment, progress, and final outcome.

The defined follow-up period encompassed the duration between the initiation of treatment with ICIs and the final visit or mortality. The onset of CIP was determined as the interval between the initial administration of ICIs and the manifestation of imaging results.

### Statistical analysis

The normality of continuous variables was evaluated using the Kolmogorov–Smirnov test. In cases where the continuous variables exhibited a normal distribution, the mean ± standard deviation was utilized to represent the values. The independent-sample t-test was employed to compare intergroup differences. For continuous data with non-normal distributions, the Wilcoxon rank-sum test was utilized to compare categorical variables between the groups, represented as medians (25th–75th percentiles). Categorical variables were presented as percentages and compared using the chi-square test. Spearman’s correlation test was utilized to examine the relationships among the study variables. The performance was accurately measured by employing the receiver operating characteristic (ROC) curve and area under the ROC curve (AUC) to quantify the results. To assess the impact of potential risk factors, univariate logistic regression was conducted, and the odds ratio (OR) and associated 95% confidence interval (CI) were calculated for each variable. To determine the independent predictors of CIP and evaluate their effects, a multivariate logistic regression analysis was conducted. The survival rates between the groups were compared using Kaplan–Meier analysis with the log-rank test. A significance level of two-sided *p* < 0.05 was deemed significant. All statistical analyses were performed using SPSS (version 23.0; IBM Corp., Armonk, NY) and R (version 4.2.2., R Software for Statistical Computing, Vienna, Austria) software.

## Results

### Derivation of the study populations

**Fig. 1:**
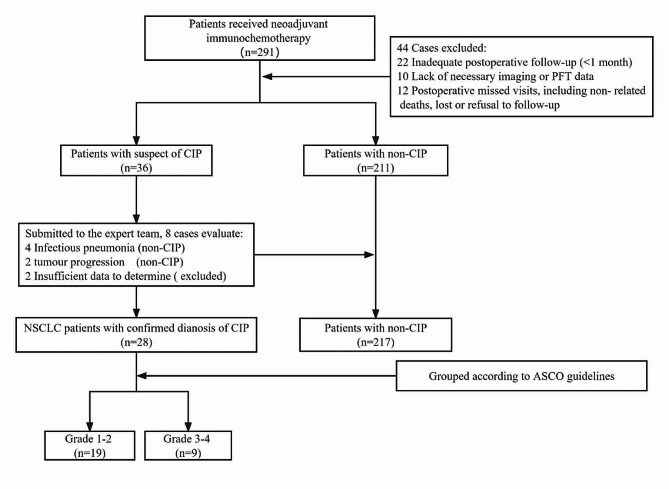
Flowchart of CIP screening Note: ASCO: American Society of Clinical Oncology; CIP: checkpoint inhibitor-associated pneumonitis; CT: computed tomography; NSCLC: non-small cell lung carcinoma; PFT: Pulmonary Function Test

Using the electronic medical record system, we identified 291 cases of NSCLC who received neoadjuvant immunochemotherapy from January 2018 to September 2022. A total of 44 cases were excluded from the study. Among these, 22 cases were excluded because of a postoperative follow-up period of < 1 month, 10 cases were excluded owing to insufficient imaging and pulmonary function data, and 12 cases were excluded owing to missed postoperative visits, which encompassed instances of death, loss of contact, and refusal to follow-up recommendations. In total, 36 patients with aberrant imaging findings during the follow-up period were assessed and assigned to an expert team for CIP diagnosis. They eliminated lesions in eight cases and confirmed lesions in 28 (of the eight patients, four were diagnosed with infection, two were found to have tumor progression, and the remaining two were excluded from the study cohort owing to the inability to make a definitive judgment). All the unqualified instances were eliminated, leaving 245 patients in the final dataset. Depending on whether CIP took place, they were split into the CIP group (11.4%) and the non-CIP group (88.6%). The duration of the follow-up period ranged from 243 to 1386 days, with a median of 511 days and a mean of 504 days (Fig. 1).

### Patient characteristics

This study had a median participant age of 56 years, with a predominantly male population. The prevalence of smoking among the participants was 66.9% (current and former). Squamous carcinoma was the most common histological type, accounting for 72.6% of cases. The majority of patients (78.0%) received platinum-paclitaxel treatment, while 49 (20.0%) received platinum-pemetrexed. The most frequently used drugs were PD-1 inhibitors (98.8%), including Sintilimab, Tislelizumab, Toripalimab, Pembrolizumab, and Camrelizumab, followed by PD-L1 inhibitors (1.2%), including durvalumab. Approximately 75.5% of patients received two courses of combined treatment, 18.0% received three, and 4.9% received four. Regarding the pathological outcomes of both cohorts, it was observed that in the non-CIP group, 87 (40.1%) patients attained pCR and 35 (16.1%) patients achieved MPR, while in the CIP group, nine (32.1%) patients achieved pCR and five (17.9%) patients achieved MPR. Of the postoperative follow-up treatments administered, 196 cases were subjected to maintenance immunotherapy, 31 cases were devoid of any postoperative treatment, 13 cases were subjected to adjuvant chemotherapy, and five cases were subjected to radiotherapy. There was no statistically significant difference observed between the two groups. The baseline features of the two groups’ demographics, comorbidities, tumor histological types, and therapies were comparable (Table 1).

**Table 1:** Baseline characteristics of the study population

	All patients (*n* = 245)	No CIP (*n* = 217)	CIP (*n* = 28)	*p*-value
Age (years)	56.0 [52.0–63.0]	56.0 [52.0–63.0]	57.0 [53.3–62.0]	0.904
Sex, n (%)				> 0.99
Female	21 (8.6)	19 (8.8)	2 (7.1)	
Male	224 (91.4)	198 (91.2)	26 (92.9)	
Smoking status, n (%)				0.453
Never	81 (33.1)	74 (34.1)	7 (25.0)	
Current or former	164 (66.9)	143 (65.9)	21 (75.0)	
Comorbidities, n (%)				
Hypertension	61 (24.9)	52 (24.0)	9 (32.1)	0.478
Diabetes	33 (13.5)	29 (13.4)	4 (14.3)	> 0.99
COPD	71 (29.0)	64 (29.5)	7 (25.0)	0.786
Course of neoadjuvant immunotherapy, n (%)				0.323
1	4 (1.6)	4 (1.8)	0 (0.0)	
2	185 (75.5)	166 (76.5)	19 (67.9)	
3	44 (18.0)	38 (17.5)	6 (21.4)	
4	12 (4.9)	9 (4.2)	3 (10.7)	
Platinum, n (%)				0.544
Carboplatin	198 (80.8)	173 (79.7)	25 (89.2)	
Nedaplatin	28 (11.4)	27 (12.4)	1 (3.6)	
Cisplatin	10 (4.1)	9 (4.2)	1 (3.6)	
None	9 (3.7)	8 (3.7)	1 (3.6)	
Chemotherapy, n (%)				0.447
Paclitaxel	191 (78.0)	168 (77.4)	23 (82.1)	
Pemetrexed	49 (20.0)	45 (20.7)	4 (14.3)	
Gemcitabine	5 (2.0)	4(1.9)	1 (3.6)	
ICI agent, n (%)				0.482
Sintilimab	79 (32.2)	67 (30.9)	12 (42.9)	
Tislelizumab	70 (28.6)	63 (29.0)	7 (25.0)	
Toripalimab	9 (3.7)	9 (4.1)	0 (0.00)	
Pembrolizumab	44 (18.0)	38 (17.5)	6 (21.4)	
Camrelizumab	34 (13.9)	32 (14.7)	2 (7.1)	
Nivolumab	6 (2.4)	6 (2.8)	0 (0.0)	
Durvalumab	3 (1.2)	2 (0.9)	1 (3.6)	
Types of ICIs, n (%)				0.306
PD-1	242 (98.8)	215 (99.1)	27 (96.4)	
PD-L1	3 (1.2)	2 (0.9)	1 (3.6)	
Histologic type, n (%)				0.839
Squamous carcinoma	178 (72.6)	158 (72.8)	20 (71.4)	
Adenocarcinoma	47 (19.2)	42 (19.4)	5 (17.9)	
Others^a^	20 (8.2)	17(7.8)	2 (10.7)	
Initial cancer stage, n (%)				0.917
II	81 (33.1)	71 (32.7)	10 (35.7)	
III	164 (66.9)	146 (67.3)	18 (64.3)	
Pathologic assessment^b^, n (%)				0.717
pCR	96 (39.2)	87 (40.1)	9 (32.1)	
MPR	40 (16.3)	35 (16.1)	5 (17.9)	
no-MPR	109 (44.5)	95 (43.8)	14 (50.0)	
LDH(U/L) L, Median (Q1,Q3) NLR, Median (Q1,Q3)	182.00 (160.00, 217.00)	182.00 (159.00, 214.00)	188.50 (175.00, 234.75)	0.119
NLR, (%)	3.20 (2.30, 4.46)	3.29 (2.31, 4.43)	2.77 (2.27, 4.67)	0.856
Pre-CT, n (%)				0.129
Normal	101 (41.2)	91 (41.9)	10 (35.7)	
Bronchitis/Emphysema	66 (26.9)	54 (24.9)	12 (42.9)	
Proliferative lesions	54 (22)	51 (23.5)	3 (10.7)	
Atelectasis	6 (2.4)	4 (1.8)	2 (7.1)	
Bronchiectasis	14 (5.7)	13 (6)	1 (3.6)	
Interstitial lung abnormality	4 (1.6)	4 (1.8)	0 (0)	
Adjuvant therapy, n (%)				0.586
No treatment	31 (12.7)	27 (12.4)	4 (14.3)	
Radiotherapy	5 (2.0)	4 (1.8)	1 (3.6)	
Immunotherapy	196 (80.0)	175 (80.7)	21 (75.0)	
Chemotherapy	13 (5.3)	11 (5.1)	2 (7.1)	

Data are presented as n (%) or medians (ranges)

Note: COPD: chronic obstructive pulmonary disease; ICI: immune checkpoint inhibitor; MPR: major pathological reactions; pCR: complete pathological reactions; PD-1: programmed cell death protein 1; PD-L1: programmed cell death receptor ligand-1; LDH: lactate dehydrogenase; NLR: neutrophil-lymphocyte ratio; Other^a^, large cell carcinoma and sarcomatoid carcinoma; Pathological Assessment^b^: Multidisciplinary recommendations for pathological assessment of lung cancer resection specimens after neoadjuvant therapy; Pre-CT: Pre-treatment Pulmonary CT Abnormalities.

### Incidence, grade, and outcomes of CIP

**Fig. 2:**
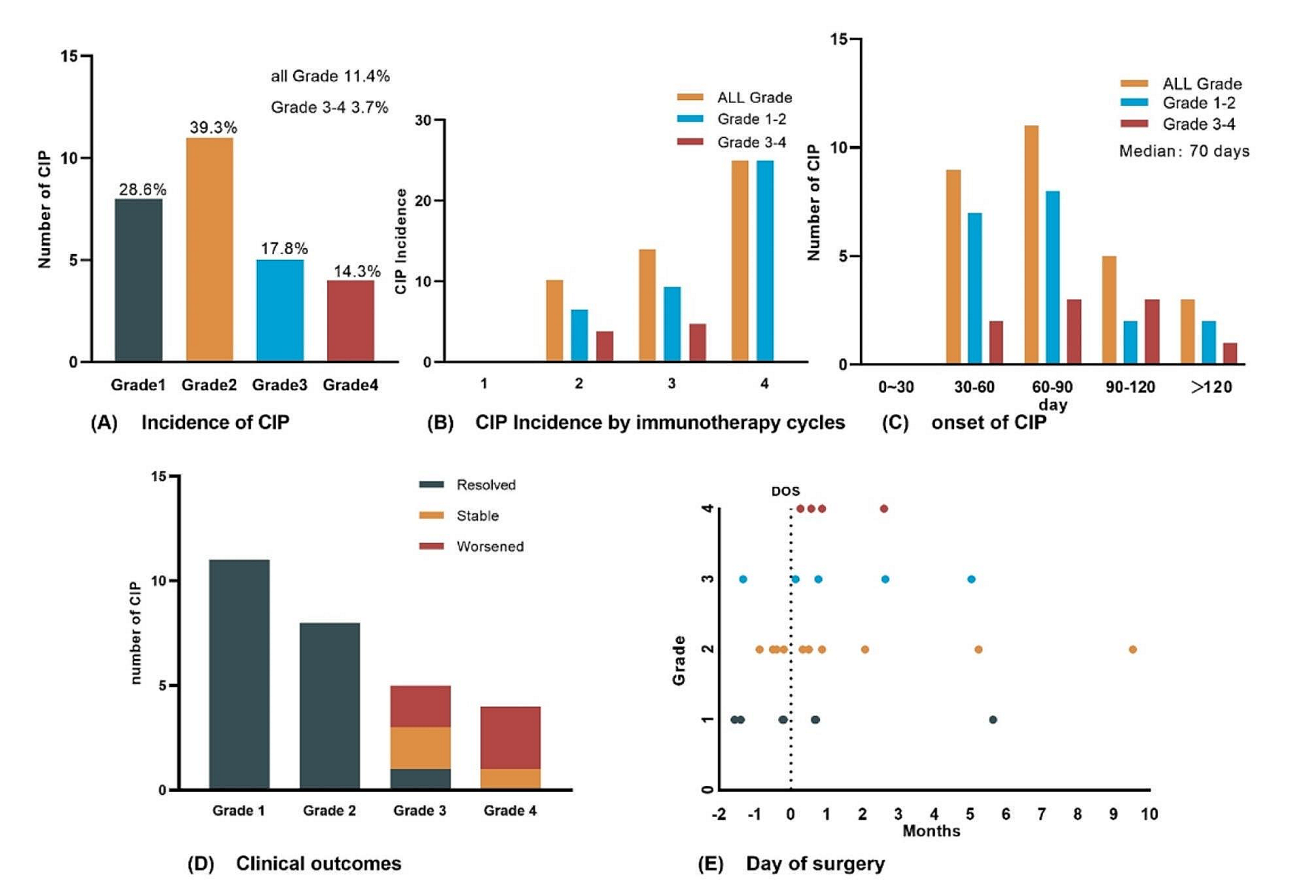
Patients with CIP were stratified according to the ASCO guideline (**A**) CIP incidence stratified by grade. (**B**) CIP incidence in relation to immunotherapy cycles. (**C**) Time of onset of CIP to the start of immunotherapy. (**D**) Clinical outcomes for different grades of CIP. (**E**) Timing of surgery and CIP occurrence Note: ASCO: American Society of Clinical Oncology; CIP: checkpoint inhibitor-associated pneumonitis; DOS: day of surgery

The frequency of CIP was 11.4% (28/245), with nine patients (3.7%) suffering from severe (grade 3–4) CIP and 19 (7.8%) having mostly mild CIP (grade 1–2). The median duration from the commencement of ICI to the manifestation of pneumonia was 70 days (range, 40–221 days), with most cases occurring within 1 month before or after surgery. The incidence of CIP for each treatment cycle is as follows: 1 cycle − 1.6%, 2 cycles − 10.3%, 3 cycles − 13.6%, 4 cycles − 25.0%. It should be noted that the cases for cycles 1 and 4 are fewer, and that the incidence rate for cycle 3 is slightly higher than for cycle 2, with similar severity proportions across cycles. All CIP patients received treatment in accordance with recommendations [6, 13], with patients graded ≥ 2 given oral or intravenous steroids, and those with mild CIP (grade 1–2) showing complete resolution by clinical criteria. Among the nine patients with grade 3–4 CIP, five deteriorated, three were stable or unchanged, and only one patient recovered from CIP. During the follow-up period, three patients died from severe CIP and three died from other causes (Fig. 2).

### Physiological and lung function indicators in the presence or absence of CIP

Our study compared the patient data before and after neoadjuvant immunochemotherapy. All collected data were obtained before surgical treatment. Patients with NSCLC and CIP had higher BMI values pre-neoadjuvant therapy (pre-BMI) (24.1 ± 3.1 vs. 22.7 ± 3.2 kg/m^2^, *p* = 0.028) and higher CRP levels post-neoadjuvant therapy (5.6 [3.1–16.1] vs. 3.8 [1.9–6.6] mg/L, *p* = 0.029). The primary distinguishing factor between the two cohorts was the FIB concentration. The CIP group had significantly higher FIB levels post-neoadjuvant therapy (4.2 [3.4–5.7] vs. 3.3 [2.8–3.8] g/L, *p* < 0.001) and a larger d-value FIB difference (0.3 [-0.9–1.7] vs. -0.6 [-1.7–0.2] g/L, *p* = 0.005).

Of the total 245 patients, 210 underwent comprehensive lung function assessments both prior to and following neoadjuvant therapy. A comparison of the FEV1/FVC% values between the two groups did not reveal any significant differences. However, discernible variations in lung function diffusion indicators were observed between the two groups subsequent to immunotherapy. Patients who developed CIP exhibited a significant decrease in both DLCO% pred and DLCO/VA% pred indicators as well as pre- and post-differences as follows: post-DLCO% pred (63.1 [53.0–72.1] vs. 71.7 [63.0–81.6], *p* = 0.001) and d-value DLCO% pred ([-12.6 ± 12.8] vs. [-6.3 ± 13.3], *p* = 0.021); post-DLCO/VA% pred (75.1 [61.5–83.5] vs. 86.8 [74.9–97.2], *p* < 0.001) and d-value DLCO/VA% pred (-15.4 [-23.1–-11.1] vs. -7.2 [-15.8–2.5], *p* < 0.001). No statistically significant variations were observed between the two groups in relation to their FBG levels or WBC counts. All data are presented in Table 2.

**Table 2:** Physiological indicators and lung function before and after neoadjuvant immunotherapy

		All patients (*n* = 245)	No CIP (*n* = 217)	CIP (*n* = 28)	*p*-value
BMI (kg/m^2^)	Pre	22.9 ± 3.2	22.7 ± 3.2	24.1 ± 3.1	0.028
	Post	22.7 [20.7–25.0]	22.3 [20.6–25.0]	23.7 [22.4–26.0]	0.109
	D-value	0.0 [-0.4–0.8]	0.0 [-0.4–0.8]	0.0 [-0.4–0.5]	0.551
CRP (mg/L)	Pre	7.7 [3.2–22.6]	7.6 [3.1–17.3]	8.8 [3.4–30.9]	0.221
	Post	3.9 [2.0–7.2]	3.8 [1.9–6.6]	5.6 [3.1–16.1]	0.029
	D-value	-2.3 [-14.9–1.3]	-2.3 [-12.7–1.3]	-2.3 [-25.8–0.8]	0.534
FBG (mmol/L)	Pre	5.3 [4.8–6.0]	5.3 [4.9–6.0]	5.0 [4.6–5.7]	0.138
	Post	5.3 [4.9–6.0]	5.3 [4.9–6.0]	5.4 [4.9–6.5]	0.733
	D-value	0.1 [-0.6–0.7]	0.1 [-0.7–0.7]	0.4 [-0.5–0.8]	0.398
WBC (10^9/L)	Pre	7.0 [5.7–8.4]	7.0 [5.7–8.4]	6.7 [5.7–8.2]	0.616
	Post	5.5 [4.5–7.4]	5.5 [4.5–7.0]	5.9 [4.3–8.8]	0.602
	D-value	-1.1 [-2.6–0.5]	-1.1 [-2.6–0.4]	-0.3 [-2.1–1.8]	0.157
FIB (g/L)	Pre	4.1 [3.1–5.1]	4.1 [3.1–5.0]	3.9 [3.0–5.4]	0.944
	Post	3.4 [2.8–3.9]	3.3 [2.8–3.8]	4.2 [3.4–5.7]	< 0.001
	D-value	-0.5 [-1.7–0.2]	-0.6 [-1.7–0.2]	0.3 [-0.9–1.7]	0.005
FEV_1_/FVC%pred	Pre	74.8 [68.3–78.7]	74.6 [68.3–78.5]	75.3 [69.9–78.8]	0.956
	Post	72.7 [66.2–77.2]	72.7 [65.9–77.2]	72.3 [69.6–77.2]	0.821
	D-value	-0.7 [-5.7–3.1]	-0.8 [-5.7–3.1]	0.1 [-3.9–4.0]	0.395
D_L_CO% pred	Pre	78.4 [67.7–86.9]	79.1 [68.7–86.9]	75.1 [65.3–84.1]	0.219
	Post	70.8 [62.3–80.7]	71.7 [63.0–81.6]	63.1 [53.0–72.1]	0.001
	D-value	-7.0 ± 13.4	-6.3 ± 13.3	-12.6 ± 12.8	0.021
D_L_CO/VA%pred	Pre	94.0 [83.1–103.8]	94.1 [83.1–104.3]	92.6 [80.2–98.2]	0.388
	Post	84.5 [73.6–95.9]	86.8 [74.9–97.2]	75.1 [61.5–83.5]	< 0.001
	D-value	-8.7 [-18.4–0.8]	-7.2 [-15.8–2.5]	-15.4 [-23.1–-11.1]	< 0.001

All data are presented as numbers (%), median (interquartile range), or mean (standard deviation)

Note: BMI: body mass index; WBC: white blood cell; FBG: fasting blood glucose; FIB: fibrinogen; CRP: C-reactive protein; Pre: pre-treatment; Post: post-treatment; D-value: difference value; PEF: peak expiratory flow; PIF: peak inspiratory flow; DLCO% pred: diffusing capacity of the lungs for carbon monoxide/predicted values; DLCO/VA% pred: diffusing capacity of the lungs for carbon monoxide by alveolar volume/predicted values; FEV1 /FVC%: forced expiratory volume in one second/ forced vital capacity ratio; FEV1: forced expiratory volume in 1 s; VC% pred: vitalcapacity/predicted values; FEV1% pred: forced expiratory volume in one second/predicted values; FVC: forced vital capacity;

### Construction of diagnostic model of CIP

**Fig. 3:**
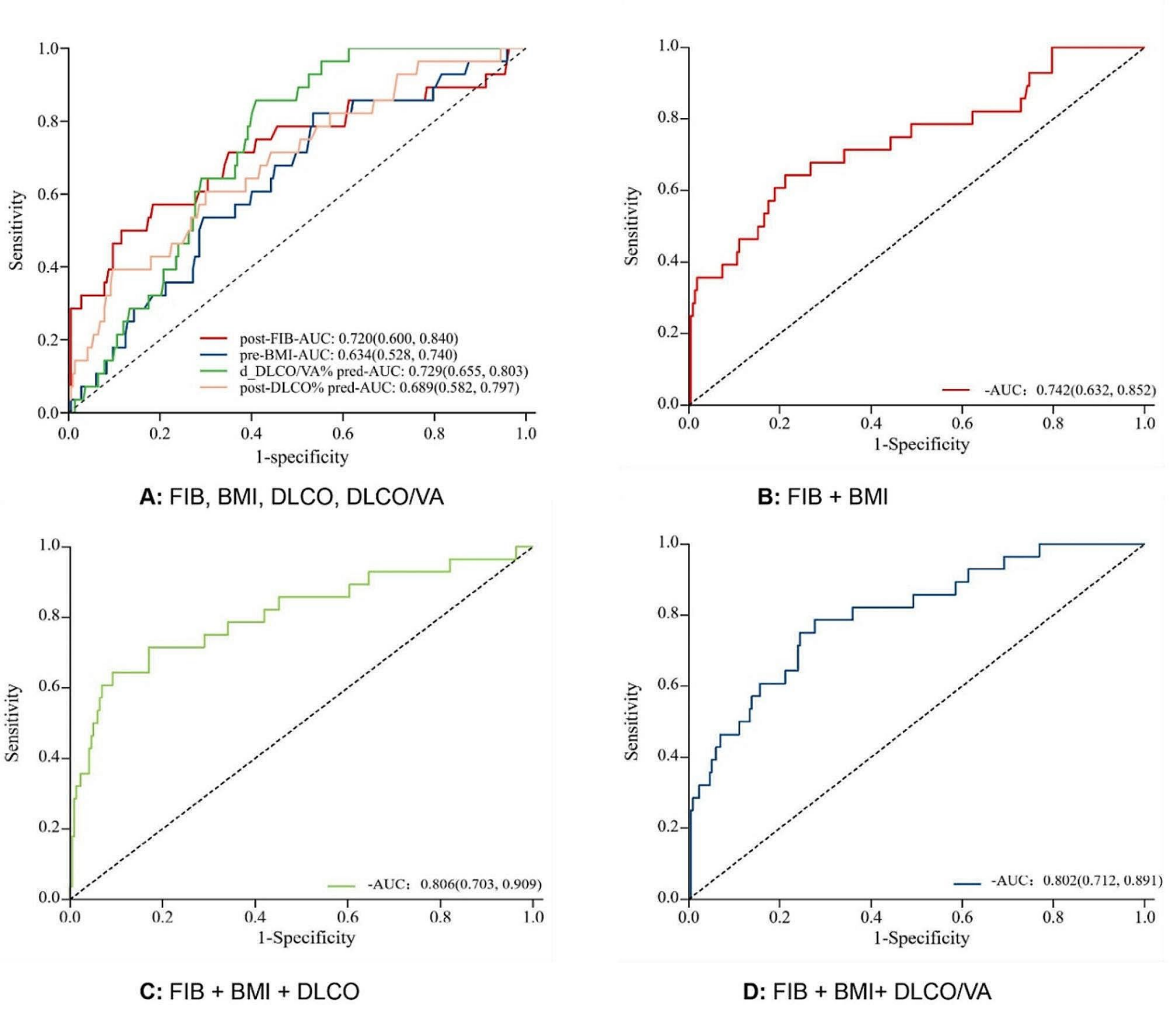
ROC curves with four features (**A**) Performance of post-FIB, pre-BMI, post-DLCO% pred and d-DLCO/VA% pred for predicting CIP. (**B**) Performance of post-FIB combined with pre-BMI for predicting CIP. (**C**) Performance of post-FIB combined with pre-BMI and post-DLCO% for predicting CIP. (**D**) Performance of post-FIB combined with pre-BMI and d-DLCO/VA% for predicting CIP Note: d-DLCO/VA% pred: d- value diffusing capacity of the lungs for carbon monoxide by alveolar volume/predicted values; post-DLCO% pred: post diffusing capacity of the lungs for carbon monoxide/predicted values; post-FIB, post-fibrinogen; pre-BMI, pre-body mass index

A ROC curve analysis was conducted to assess the efficacy of these correlators in predicting CIP at an early stage. Using − 10.05 as the cutoff value, D-DLCO/VA% pred obtained a sensitivity of 59.1% and a specificity of 85.7% in warning of the occurrence of CIP, with an AUC of 0.729 (95% CI, 0.655–0.803; *p* < 0.001). The other two predictors with higher AUCs were post-FIB, with the best cutoff value of 3.955 g/L, a sensitivity of 57.1%, a specificity of 81.6%, and an AUC of 0.720 (95% CI, 0.600–0.840, *p* < 0.001), and post-DLCO% pred, with a best cutoff value of 64.85, sensitivity of 70.0%, specificity of 60.7%, and AUC of 0.689 (95% CI, 0.582–0.797, *p* < 0.001). Although the sensitivity of pre-BMI (82.1%; 95% CI, 0.680–0.963) was high, the specificity was low (46.5%; 95% CI, 0.399–0.532), and the best cutoff value was 22.0. Figure 2B–D demonstrate the amalgamated prediction curves for the three assessed variables (FIB + BMI, FIB + BMI + DLCO, FIB + BMI + DLCO/VA). The ROC models utilized for combining these variables were efficacious in augmenting the AUCs of the combined variables, resulting in a corresponding increase in their predictive values. Notably, the utilization of all three variables concurrently yielded AUCs of 0.806 and 0.802, respectively, which can be deemed satisfactory (Fig. 3).

### Kaplan–Meier analysis of incidence of CIP

**Fig. 4:**
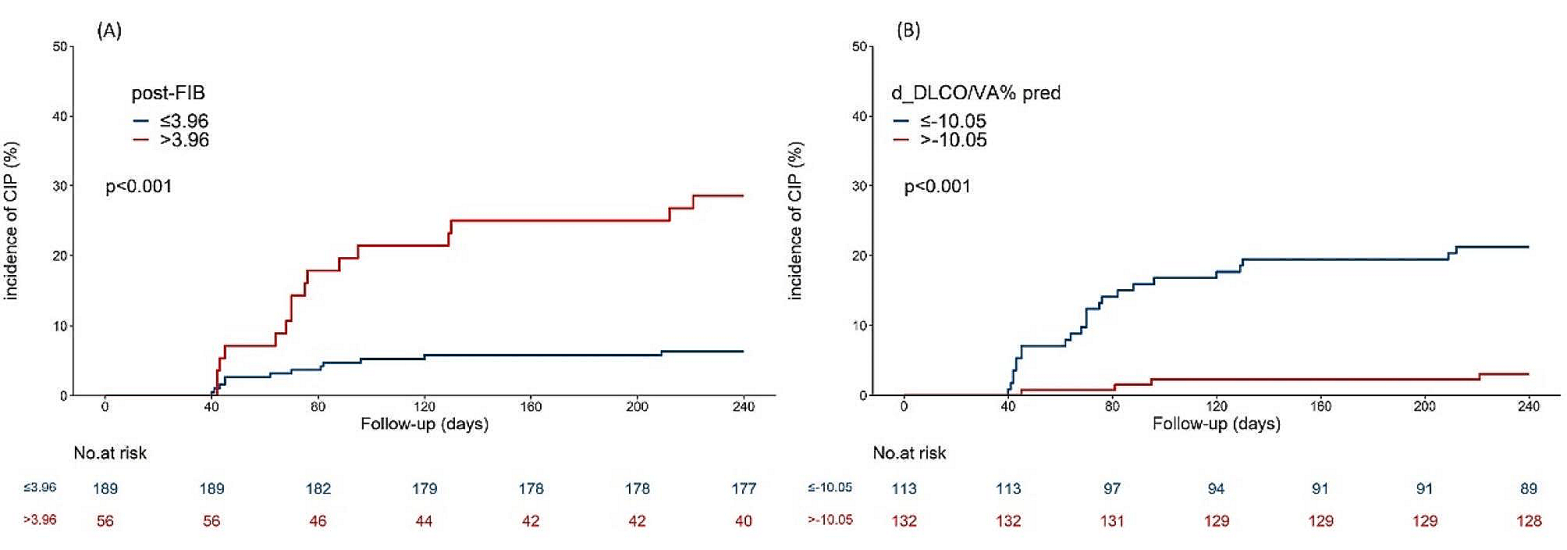
The impact of the FIB and DLCO/VA% pred on incidence of CIP was evaluated using the Kaplan–Meier method (*p*-value according to the log-rank test) (**A**) Patients with high FIB expression tended to have a higher incidence of CIP compared with those with low FIB expression. (**B**) CIP is more likely to occur in cohorts with a large absolute DLCO/VA% pred difference Note: CIP: checkpoint inhibitor-associated pneumonitis; FIB: fibrinogen; post-FIB: post-fibrinogen; d-DLCO/VA% pred: d- value diffusing capacity of the lungs for carbon monoxide by alveolar volume/predicted values

To investigate the potential correlation between post-FIB and d-DLCO/VA% pred levels and the occurrence of CIP, the patient cohort was stratified into the low-FIB (< 3.96 g/L) and high-FIB (≥ 3.96 g/L) groups as well as into the low-d-DLCO/VA% pred (<-10.05) and high-d-DLCO/VA% pred (≥-10.05) groups. Subsequently, a Kaplan–Meier survival analysis was conducted, which showed that patients with high FIB expression exhibited a greater likelihood of developing CIP in comparison to those with low FIB expression. Additionally, the group with a larger d-DLCO/VA% pred was found to be more susceptible to CIP (log-rank test, *p* < 0.001) (Fig. 4).

### Univariate and multivariate analyses for predicting development of CIP

We analyzed two patient groups and found no significant differences in factors like age, gender, smoking history, pulmonary diseases, diabetes, LDH, NLR and pre-treatment pulmonary CT abnormalities. However, post-FIB was identified as a significant risk factor for CIP development (OR, 2.21; 95% CI, 1.53–3.35; *p* < 0.001). In our univariate analysis of key pulmonary function parameters, such as FEV1, VC, PEF, and PIF, as well as their differences before and after treatment, no significant differences were observed. However, post-DLCO% pred exhibited a statistically significant improvement (OR, 0.95; 95% CI, 0.92–0.98; *p* = 0.001). Similarly, significant enhancements were observed in the post-DLCO/VA % pred (OR, 0.95; 95% CI, 0.93–0.98; *p* < 0.001), and d-DLCO/VA % pred (OR, 0.96; 95% CI, 0.93–0.98; *p* = 0.001). In addition, the adverse factors associated with CIP included pre-BMI, pre-CRP level, and d-DLCO% predicted. All data are presented in Table 3.

**Table 3:** Univariate analyses to determin risk factors for CIP

Variable	Category	Univariate		
		OR	95%CI	*p*-value
Sex	Male	1.24	0.34–8.11	0.775
Age		0.99	0.95–1.05	0.962
Smoke	Yes	1.55	0.66–4.09	0.338
COPD	Yes	0.80	0.30–1.89	0.622
Diabetes	Yes	1.02	0.46–2.28	0.954
Histology	Adenocarcinoma	0.94	0.30–2.48	0.908
LDH		1.01	1.00-1.01	0.059
NLR		0.97	0.80–1.11	0.719
Pre-CT	Bronchitis/Emphysema	2.02	0.82–5.10	0.127
	Proliferative lesions	0.54	0.12–1.84	0.359
	Atelectasis	4.55	0.58–26.63	0.102
BMI, (kg/m^2^)	Pre	1.15	1.02–1.30	0.028
	Post	1.11	0.99–1.25	0.087
	D-value	0.97	0.85–1.18	0.702
CRP, (mg/L)	Pre	1.01	1.00-1.02	0.007
	Post	1.01	0.99–1.02	0.282
	D-value	0.99	0.98-1.00	0.032
WBC, (10^9/L)	Pre	0.95	0.77–1.15	0.591
	Post	1.09	0.93–1.25	0.267
	D-value	1.11	0.96–1.27	0.159
FIB, (g/L)	Pre	1.11	0.84–1.44	0.466
	Post	2.21	1.53–3.35	< 0.001
	D-value	1.47	1.10-2.00	0.012
FEV1% pred	Pre	1.00	0.98–1.03	0.746
	Post	0.99	0.97–1.02	0.571
	D-value	0.99	0.96–1.01	0.338
VC% pred	Pre	1.00	0.98–1.04	0.479
	Post	0.99	0.96–1.01	0.334
	D-value	0.97	0.94-1.00	0.081
PEF, (L/min)	Pre	1.05	0.83–1.31	0.702
	Post	1.11	0.88–1.40	0.380
	D-value	1.11	0.81–1.54	0.515
PIF, (L/s)	Pre	1.07	0.82–1.37	0.621
	Post	1.15	0.89–1.48	0.269
	D-value	1.11	0.83–1.50	0.497
FEV_1_/FVC% pred	Pre	0.99	0.94–1.04	0.616
	Post	1.01	0.97–1.06	0.646
	D-value	1.03	0.98–1.09	0.319
D_L_CO % pred	Pre	0.99	0.96–1.01	0.253
	Post	0.95	0.92–0.98	0.001
	D-value	0.97	0.94–0.99	0.022
D_L_CO/VA % pred	Pre	0.99	0.97–1.01	0.412
	Post	0.95	0.93–0.98	< 0.001
	D-value	0.96	0.93–0.98	0.001

BMI, body mass index; CRP, C-reactive protein; WBC, white blood cell; FIB, fibrinogen; PEF, peak expiratory flow; PIF, peak inspiratory flow; FVC, forced vital capacity; VC% pred, vitalcapacity/predicted values; FEV1% pred, forced expiratory volume in one second/predicted values; LDH, lactate dehydrogenase; NLR, neutrophil-lymphocyte ratio; Pre-CT: Pre-treatment Pulmonary CT Abnormalities; DLCO% pred, diffusing capacity of the lungs for carbon monoxide/predicted values; DLCO/VA% pred, diffusing capacity of the lungs for carbon monoxide by alveolar volume/predicted values; Pre: pretherapy; Post: post-treatment; D-value: difference value

The forward stepwise method was used for a multivariate logistic regression analysis. To prevent multicollinearity and overfitting, we split the risk factors post-DLCO% pred and d-DLCO/VA% pred into two combinations for analysis. Both models showed that pre-BMI, post-FIB, post-DLCO% pred, and d-DLCO/VA% pred were independent risk factors for CIP. All data are presented in Table 4.

**Table 4:** Potential factors associated with CIP: results of multivariate regression

	OR	95%CI	*p*-value
*Model 1*			
pre-BMI	1.27	1.10–1.47	0.001
post-FIB	2.09	1.43–3.24	< 0.001
post-DLCO % pred	0.94	0.91–0.98	0.001
*Model 2*			
pre-BMI	1.19	1.04–1.37	0.013
post-FIB	2.25	1.56–3.40	< 0.001
D-DLCO/VA % pred	0.96	0.93–0.99	0.003

BMI, body mass index; FIB, fibrinogen; DLCO% pred, diffusing capacity of the lungs for carbon monoxide/predicted values; DLCO/VA% pred, diffusing capacity of the lungs for carbon monoxide by alveolar volume/predicted values; Pre: pretherapy; Post: post-treatment; D-: difference value

## Discussion

The ICIs into the neoadjuvant therapy for early-stage, resectable NSCLC has markedly transformed the landscape of treatment. It has significantly enhanced the surgical resection rates, reducing recurrences rates, and significantly prolonged patients’ survival, moving us closer to achieving potential curative outcomes [14, 15]. However, this breakthrough is not without challenges. The intricate dynamics between the immune system and the tumor microenvironment, while serving as a therapeutic target, also lead to a heightened occurrence of of irAEs [16]. This complexity underscores the need for precision in treatment planning and the imperative for identifying reliable biomarkers that can predict irAEs, enabling clinicians to finely tune the therapeutic benefits against the risks, navigating towards the most favorable outcomes for patients. This study is focused on investigating the incidence, risk factors, and clinical outcomes of CIP in patients with resectable NSCLC receiving neoadjuvant immunochemotherapy. Previous clinical studies have reported CIP incidence rates of less than 5% in advanced NSCLC contexts [17, 18]. However, the incidence in the real world is often greater, at 14.5% for monotherapy and 19% for combination medications [19, 20]. The NADIM trial study revealed a notable frequency of CIP in patients with NSCLC who underwent neoadjuvant immunotherapy [21]. In our study on neoadjuvant chemoimmunotherapy, the incidence of CIP was 11.4% and the proportion of grade ≥ 3 CIP cases was 3.7%, which was lower than that reported in these real-world studies of patients with advanced NSCLC. Mild CIP accounted for 67.9% of the total, and they all improved after treatment. We attribute this result to young age and good physiological status, which are reportedly associated with less severe CIP outcomes [22]. However, the surgery itself is a risk factor for interstitial pneumonitis [23]. In our study, we found that the majority of severe CIP cases were observed postoperatively. The median duration until the onset of CIP was 70 days, which aligns with the outcomes of prior studies [24, 25]. The majority of CIP occurred between 1 month before and after surgery, which appears to coincide with the immunotherapy cycle. Therefore, for patients treated with neoadjuvant immunochemotherapy, follow-up within 1 month before and after surgery is crucial.

The ASCO Clinical Oncology guidelines recommend that maintenance therapy with ICIs be tolerated in advanced NSCLC until disease progression or intolerance [26]. However, the course of neoadjuvant immunotherapy is relatively short and inconclusive. Inadequate neoadjuvant treatment courses may not be sufficient to induce the therapeutic effects of ICIs. However, considering the progression of lung cancer, extending the course of immunotherapy and delaying surgery may pose significant risks to the patient. Most previous clinical trials on neoadjuvant immunotherapy have used 2–4 courses of single-agent or combination chemotherapy [27–29]. The MPR of cycles increased by 14.5% compared to that of the two cycles [30]. Our observation revealed that the occurrence of CIP was slightly elevated when patients underwent three cycles of neoadjuvant immunochemotherapy treatment compared to the corresponding after undergoing two cycles. Furthermore, despite a notable rise in the occurrence of CIP following the administration of four cycles of immunization, all patients exhibited only a mild grade of CIP (Fig. 2B). Our results seem to indicate that the incidence of CIP would potentially rise in tandem with the number of immunotherapy cycles administered. However, further sample expansion is needed to validate these results.

Currently, clinicians rely heavily on respiratory symptoms and signs presented by patients combined with chest imaging to diagnose CIP. Smoking history, previous lung disease (i.e., chronic obstructive pulmonary disease, interstitial lung disease), histological type, radiotherapy, and combination with ICIs or other drugs have been shown to be risk factors for the occurrence of CIP [9, 31, 32]. However, only a few studies have investigated neoadjuvant immunotherapy. Our study focused on a population undergoing neoadjuvant immunochemotherapy and found that post-FIB, post-DLCO% pred, and d-DLCO/VA % pred, pre-BMI were independent risk factors for CIP.

A previous study showed that increased FIB level is a predictive factor of obstructive and restrictive lung diseases [33]. Similarly, in the present study, patients with elevated post-FIB levels had an increased risk of developing CIP. FIB is an acute-phase protein, whose level increases during any form of inflammation. Numerous investigations have documented the significance of elevated plasma fibrinogen levels in the prognostication of lung cancer [34, 35]. The inhibition of the PD-1 pathway has the potential to augment T-cell responses against tumors. Nevertheless, the targeted intensification of T-cell responses and the elevation of inflammatory cytokine levels may contribute to the development of irAEs [11, 36]. Interleukin (IL)-6, IL-2, IL-17, IL-35, and CRP levels are reportedly higher in patients with NSCLC who develop CIP [11, 37]. All these factors contribute to high FIB expression. In our study, when analyzing the post-FIB levels (3.955 g/L) separately, we observed a 22% elevated risk of all-cause mortality. Both univariate and multivariate analyses demonstrated that elevated FIB levels were significant independent risk factors for CIP.

Lung diffusing capacity for carbon monoxide is a sensitive indicator of pulmonary fibrosis and predicts changes in lung imaging findings [38]. As guidelines recommend regular DLCO measurement to detect CIP, in patients with NSCLC receiving immunotherapy, clinicians must frequently explore the decline in DLCO. However, there are no specific details regarding the indicators and periodicity of diffusion function monitoring. In our study, we recorded the baseline lung function and selected DLCO% pred and d-DLCO/VA% pred as the main indices to avoid individual differences. An earlier study on the adverse effects of neoadjuvant chemotherapy, particularly the combination of carboplatin and paclitaxel, showed a reduction in DLCO [39]. Another retrospective study found that patients with NSCLC receiving neoadjuvant chemotherapy, who developed diffusion dysfunction, were more likely to have an increased risk of pulmonary resection, such as pneumonia (3/132; 2%) [40]. We also observed a variable decrease in DLCO% pred and d-DLCO/VA% pred in patients with CIP. When the d-DLCO/VA% pred decreased by 10.05%, all-cause mortality was significantly higher. Our results suggest that these two parameters can be used as warning indicators of CIP occurrence and are easily carried out in clinical practice.

Being overweight is also an independent risk factor for CIP in our study and is thought to be associated with chronic inflammation. A prior study examining patients treated with pembrolizumab demonstrated that the incidence of irAEs rose by 9% with each incremental increase of 1 kg/m^2^ in the BMI values [41]. Other studies have shown an increased risk of high-grade irAEs in patients with BMI values > 25 kg/m^2^ [42]. The identification of elevated levels of inflammatory factors in both the serum and bronchoalveolar lavage fluid of individuals with CIP serves as further evidence of their role in the pathogenesis of this condition [43, 44]. It can be hypothesized that inflammatory factors, released from the adipose tissue in patients who are overweight, are also involved in CIP.

This study had some limitations. First, this was a single-center retrospective study. The low incidence rate of CIP results in a limited number of patients and lacks another cohort to verify our findings. Second, the clinical manifestations of CIP are non-specific. Relying solely on respiratory symptoms and lung images may lead to clinical misdiagnoses or missed diagnoses.

## Conclusions

This study provides early warning indicators for CIP occurrence in patients with NSCLC receiving neoadjuvant immunochemotherapy. Early monitoring of changes in plasma fibrinogen and lung diffusion function during neoadjuvant immunotherapy can help identify high-risk patients for severe immune-related pneumonia and adopt timely intervention strategies.

## Data Availability

The datasets used or analyzed in the current study are available from the corresponding author upon reasonable request.

## Abbreviations

ADLs: Activities of daily living
BMI: Body mass index
BMI: Body mass index
CI: Confidence interval
CIP: Checkpoint inhibitor associated pneumonitis
CRP: C-reactive protein
CT: Computed tomography
DFS: Disease-free survival
FBG: Fasting blood glucose
FIB: Increased fibrinogen
ICIs: Immune checkpoint inhibitors
irAEs: Immune-related adverse events
MPR: Major pathological reactions
NSCLC: Non-small cell lung cancer
OR: Odds ratio
OS: Overall survival
pCR: Complete pathological reactions
PD-1: Death protein 1
PD-L1: PD receptor ligand-1
ROC: Receiver operating characteristic curve
ROC: Receiver operating characteristic
TNM: Tumor node metastasis
WBC: White blood cell
